# Von Willebrand's disease: case report and review of literature

**DOI:** 10.11604/pamj.2017.27.147.12248

**Published:** 2017-06-29

**Authors:** Hanae Echahdi, Brahim El Hasbaoui, Mohamed El Khorassani, Aomar Agadr, Mohamed Khattab

**Affiliations:** 1 Center of Hematology and Oncology Paediatrics, Children's Hospital, Faculty of Medicine and Pharmacy, University Mohammed V Rabat, Morocco; 2 Department of Pediatrics, Military Teaching Hospital Mohammed V, Faculty of Medicine and Pharmacy, University Mohammed V, Rabat, Morocco

**Keywords:** Von Willebrand disease, Von Willebrand factor, factor VIII, platelets, hemorrhage

## Abstract

Von Willebrand Disease (VWD) is the most common human inherited bleeding disorder due to a defect of Von Willebrand Factor (VWF), which a glycoprotein crucial for platelet adhesion to the subendothelium after vascular injury. VWD include quantitative defects of VWF, either partial (type 1 with VWF levels < 50 IU/dL) or virtually total (type 3 with undetectable VWF levels) and also qualitative defects of VWF (type 2 variants with discrepant antigenic and functional VWF levels). The most bleeding forms of VWD usually do not concern type 1 patients with the mildest VWF defects (VWF levels between 30 and 50IU/dL). Von willebrand factor is a complex multimeric protein with two functions: it forms a bridge between the platelets and areas of vascular damage and it binds to and stabilizes factor VIII, which is necessary for the clotting cascade. By taking a clinical history of bleeding (mucocutaneous bleeding symptoms suggestive of a primary haemostatic disorder, a quantitative or qualitative abnormality of VWF is possible) it is important to think about VWD and to make the appropriate diagnosis. If the VWD is suspected diagnostic tests should include an activated partial thromboplastin time, bleeding time, factor VIII: C Ristocetin cofactor and vWF antigen. Additional testing of ristocetin induced plattlet adhesion (RIPA) the multimeric structure and collagen binding test and genanalysis allow diagnosing the different types of von. Willebrand Disease. The treatment of choice in mild forms is the synthetic agent desmopressin. In patients with severe type 1, type 2B, 2N and type 3 or in people who do not response to desmopressin, the appropriate treatment is a factor VIII concentrate that is rich of VWF. We report a case of infant in 27-month-old boy who had been referred due to haemorrhagic shock. His birth histories, his familie's social history and developmental milestones were unremarkable. He was born at full term with no antenatal or perinatal complications. Prior to the symptoms, the child was on a normal diet and was thriving appropriately. The child presented one days before his admission trauma to the inner face of the lower lip that caused an external acute bleeding loss. The laboratory data showed unfortunately, the most severe form of Von Willebrand's Disease; Type 3. The management was based on erythrocyte and fresh-frozen plasma (FFP) transfusions with administration of factor VII with good evolution.

## Introduction

Von Willebrand disease (VWD) is usually reported to be “the most common human inherited bleeding disorder.” Actually found in approximately 1% of the general population [1]. Originally described by the Finish physician Erik von Willebrand in 1926. VWD is due to quantitative deficiencies and/or qualitative defects in von Willebrand factor (VWF), a complex plasma protein with multiple functions, which overall contribute to the formation of a platelet thrombus at sites of injury to help prevent blood loss [2]. VWF accomplishes this major haemostasis function by anchoring platelets to sites of vascular injury (primary haemostasis function), as well binding to factor VIII (FVIII), thus protecting FVIII from degradation and delivering it to sites of vascular injury (thereby facilitating secondary haemostasis). VWF binds to platelets via several receptors, most notably glycoprotein Ib(GPIb), but also GPIIb/IIIa. VWF also binds to subendothelial matrix components, most notably collagen and in this way facilitates anchoring of platelets to damaged endothelium by forming an adhesive bridge. The peculiar structure of VWF, in terms of its size and formation of multimers has particular relevance here, as the larger VWF molecules facilitate better anchoring of platelets and thus thrombus formation and prevention of bleeds. This a heterogeneous quantitative or qualitative deficiency in the von Willebrand factor (vWF) may also be associated with a concomitant decrease in factor VIII levels (FVIII), since vWF serves as the carrier protein for FVIII [3]. It is well recognized that deficiency of VWF results in a bleeding disorder that varies in severity according to the degree of deficiency and the specific characteristics of the molecule and which may have features of both primary and secondary haemostatic defects. Clinical expression of VWD is usually mild in type 1, with increasing severity in type 2 and type 3. In general, the severity of bleeding correlates with the degree of the reduction of FVIII. Mucocutaneous bleeding (epistaxis especially during childhood, easy bruising) is a typical, prominent manifestation of the disease and may affect the quality of life. However, the rate of spontaneous bleeding may be low even in patients with severe VWF deficiency [4]. We report the observation of a child admitted to the emergency department for a state of haemorrhagic shock, revealing a severe defect in von villebrand factor.

## Patient and observation

We report the 27-month-old man who had been referred due to haemorrhagic shock. Pregnancy and birth history were unremarkable. The boy had been born full-term (39 weeks) with a birth weight (3200g). No parental consanguinity was observed. He was on exclusively breast-fed, there were no incidents during vaccinations, food diversification was started at 6 months old, his weight, length and psychomotor development were within the normal range. He was until yet not circumcised. Prior to the symptoms, the child was described as a good eater, was on a normal diet and was thriving appropriately. The boy was admitted in our department for hemorrhagic shock, he was lethargic, very pale with profound hypotonia, tachycardic, tachypnea and Oliguria at the presentation, the capillary refill time (CRT) was > 3s. Blood was oozing from a wound at the inner surface of the lower lip, the gums appeared otherwise healthy. No other abnormal findings were evident on physical examination (there were no organomegaly and no joint disease (Hemarthrosis). The child had one days before his admission a trauma to the inner face of the lower lip that caused an external acute bleeding loss. The initial step of resuscitation was; immediately tried to stop the source of hemorrhage by manual compression, to restore circulating blood volume by administration of hypertonic salt solutions through large intravenous access calibre with providing an adequate oxygenation. Blood count pronounced: hemoglobin (Hb) level at 3 g/dL; leukocyte and platelet count were normal. Blood chemistry showed: Prothrombin Time (PT) and fibrinogen level were normal while activated partial thromboplastin time (PTT or APTT) was elongated. On the basis of the clinical presentation of hemorrhage, a tentative diagnosis of vWD had been made. This diagnosis was supported by the normal platelet count and the results of the coagulation panel. Following erythrocyte and fresh-frozen plasma (FFP) transfusions, administration of factor VII (Novoseven) was initiated at the admission. Less than 2 hr after transfusion began, the child appeared pinker and the oral bleeding had stopped. Three hours after transfusion, vital signs were within normal limits; the heart rate had decreased to 75 bpm and the respiratory rate was 26 bpm. The rest of biologic data was reported on day 4 and showed normal factor IX level but factor VIII level was low at 16.6%. The result of Factor Von Willebrand: Ag assay was very low at 1.8% that confirmed a severe form of Von Willebrand disease (type 3). The child was discharged with recommendation of relatif sedentary lifestyle in combination with prophylactic therapy in minor surgery including dental work.

## Discussion

Von Willebrand factor (VWF) is a large multimeric glycoprotein crucial for primary hemostasis and for coagulation. It is synthesized as a monomeric protein, secreted into plasma and their size is regulated by a specific cleaving-protease named ADAMTS13 (a disintegrin and metalloproteinase with thrombospondin type 1 repeats, member 13) [5]. VWF is synthesized by endothelial cells and megakaryocytes. The gene coding for VWF (VWF) has been cloned and located at chromosome 12p13.2. It is a large gene of approximately 178 kilobases and containing 52 exons. VWD is a complex genetic disorder in which three subtypes have been described. The simplified classification of VWD proposed Sadler is still in common use. Despite the potential for reclassification based on molecular defects, there has been a reluctance to move to a more complex taxonomy; only minor qualifications were introduced when last reviewed. Table 1 summarizes how the classification is currently applied [6]. These include both quantitative and qualitative defects. Type 1 accounts for 70% of cases and is the mildest form of the disease. Type 1 cases are caused bya partial deficiency of VWF. Type 2 cases are more difficult to diagnose due to the qualitative nature of the defect. These defects range from absence of certain protein multimers for binding during hemostasis to improper binding and decreased affinity. This Type 2 sub-group accounts for approximately 20-30% of cases. Qualitative VWD type 2 is further divided into four variants: 2A, 2B, 2N and 2M, based on the characteristics of the dysfunctional vWF. The type of mutation affecting the vWF locus forms the basis for classification of most type 2 VWD variants. Fortunately, the most severe form, Type 3, is rare. It accounts for 5% of cases overall. However, in some Swedish communities with prevalent disease, 1/ 200,000 people may have the severe form [7]. The diagnosis of VWD is based on clinical and biological information. When patients present with mucocutaneous bleeding symptoms suggestive of a primary haemostatic disorder, a quantitative or qualitative abnormality of VWF is a possible cause or contributory factor. During the initial assessment it is important to remember that bleeding histories can be subjective and the disease characteristics can take time to evolve. A medical health history is important to help determine if other relatives have been diagnosed with a bleeding disorder or have experienced symptoms.

**Table 1:** Classification of VWD

TYPE	Description	Comments	Inheritance
1	Partial quantitative deficiency of VWF	Includes *VWF* mutations causing rapid VWF clearance (e.g. VWF Vicenza) and requires function: antigen ratio >0.6	Mostly autosomal dominant inheritance when VWF < 0.3 IU/ml. Mutations of *VWF* in kindred with levels > 0.3 IU/ml show variable penetrance
2	Qualitative VWF defects		
2A	Decreased VWF-dependent platelet adhesion with selective deficiency of high-molecular-weight multimers	Some controversy exists regarding classification of *VWF* mutations associated with subtle reductions in HMW multimers	Mostly autosomal dominant
2B	Increased affinity for platelet GPIb	Should be distinguished from PT-VWD, using either platelet agglutination tests or genetic testing. Cases with normal VWF multimer and platelet count have been described	Autosomal dominant
2M	Decreased VWF-dependent platelet adhesion without selective deficiency of HMW multimers	This also includes defects of VWF collagen binding. May be combined quantitative/qualitative defect	Autosomal dominant
2N	Markedly decreased binding affinity for FVIII	Should be distinguished from mild haemophilia A	Reduced VWF: FVIII binding defects are more commonly identified in a compound heterozygote state with a VWF null allele rather than the classical homozygous form
3	Virtually complete deficiency of VWF	Equivalent to < 0.03 iu/ml in most assays	Autosomal recessive, frequent null *VWF* alleles. Bleeding symptoms in 26 - 48% of obligate carriers

VWD, von Willebrand disease; PT-VWD, platelet type pseudo-VWD; VWF, von Willebrand factor; *VWF*, VWF gene; FVII, factor VIIIGPIb, glycoprotein Ib; HMW, high molecular weight

**Figure 1:**
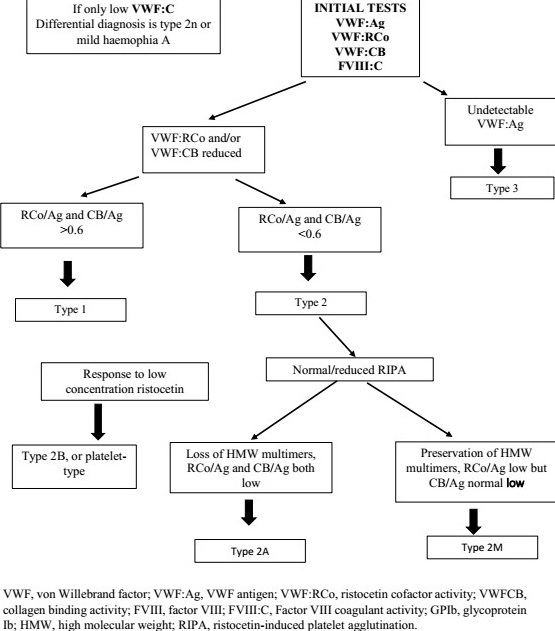
An algorithm for the investigation of suspected VWD

Screening assays include classically activated partial thromboplastin time (aPTT), platelet count and closure time (PFA-100 analyzer). Second-level-specific VWF assays are crucial to diagnose VWF deficiency and they include the measurement of FVIII activity (FVIII:C), VWF antigen (VWF:Ag) and VWF ristocetin cofactor activity (VWF:RCo) allowing the calculation of ratios (VWF:RCo/VWF:Ag and FVIII:C/VWF:Ag) and the measurement of ristocetin-induced platelet aggregation (RIPA). Third-level VWF assays are devoted to a better characterization of VWD types and they include structural assays (VWF multimers analysis, VWF propeptide (VWFpp)) and functional assays (VWF binding to platelet GPIb, to collagen (VWF:CB) and to FVIII (VWF:FVIIIB)) [8, 9]. The algorithm diagnosis of von Willebrand disease is summarizes in Figure 1 [6]. The diagnosis of VWD type and subtype is crucial for many purposes: to make a differential diagnosis between VWD 2N and hemophilia A or between VWD 2B and a platelet disorder named “pseudo VWD or platelet type VWD,” [10] to predict the response to desmopressin [11] or the kinetic of VWF during pregnancy, to anticipate the risk of allo-immunization in patients with type 3 VWD [12] and to help for genetic counseling [13]. When a diagnosis of VWD is made it is appropriate to test first degree relatives with or without a positive bleeding history. In this circumstance, a presumptive diagnosis of VWD may be made on the basis of laboratory findings alone. For patients with “low VWF” (0.3-0.5 iu/ml), family testing may be justifiable depending on bleeding and family history. The severity of the bleeding tendency is usually proportional to the degree of the primary deficiency of VWF and to that of the secondary deficiency of FVIII, as VWF is the carrier of FVIII in circulating plasma [14, 15]. Thus, in VWD the aim of therapy is to correct the dual defect of hemostasis, i.e the abnormal platelet adhesion-aggregation and the abnormal intrinsic coagulation due to low FVIII levels. In management of VWD there are available therapies to correct haemostasis comprise the non-concentrate therapies tranexamic acid and desmopressin or concentrates containing either high purity VWF alone or intermediate purity concentrates containing FVIII-VWF; the pharmacology and clinical use of desmopressin to temporarily elevate FVIII and VWF levels by releasing endothelial stores have been extensively, because of its clinical utility and the wide variation in response, a trial of desmopressin should be considered in all patients with type 1, type 2A, 2M and 2N VWD who do not have a contraindication to its use. It is also useful in patients with bleeding and low VWF as a risk factor ("low VWF").

In type 2B a transient thrombocytopenia frequently follows desmopressin administration [16]. This has been regarded as a reason for contraindication and although no harmful effects have been reported, the therapeutic response is usually poor and desmopressin is not recommended for type 2B VWD [17]. Desmopressin is relatively contraindicated in children < 2 years old. If after careful consideration, it is to be used in this age group then fluid restriction, avoidance of hyponatraemic solutions and close monitoring of serum electrolytes and urine output for at least 24 hr after administration is advised. Tranexamic acid administered topically, as a mouthwash, orally or parenterally remains a useful therapy for minor bleeding or surgery (beginning prior to the procedure) either on its own or as an adjunctive therapy to desmopressin or concentrates [18]. VWF-containing concentrates a number of plasma-derived concentrates containing VWF are available for replacement therapy in patients whose desmopressin response is inadequate for the relevant bleeding episode or surgical procedure [19]. For haemostatic activity, the relevant characteristics of these concentrates are the multimeric composition of the VWF and the amount of FVIII contained per unit of VWF. A VWF:RCo/VWF: Ag ratio close to 1 is desirable because it indicates the VWF has normal multimeric structure and adhesive function, but the appropriate amount of FVIII is debatable and will vary according to the circumstance. It's recommended for treatment of acute bleeding or emergency surgery, a VWF-FVIII concentrate or a combination of high purity FVIII and high purity VWF concentrates should be used. Treatment of acute bleeding episodes in VWD such as epistaxes, gum bleeding and menorrhagia. Post-traumatic bleeding can also occur and type 3 patients can develop spontaneous joint or muscle bleeding. When any of these are frequent, self-administration of desmopressin and tranexamic acid can be helpful [20]. If the patient is non-desmopressin responsive, then acute treatment transfusion and secondary prophylaxis using concentrates should be considered. When bleeding persists despite apparently normal plasma levels of VWF activity, platelet transfusion may be helpful. Platelet transfusion is also the treatment of choice in platelet-type VWD pseudo (PT-VWD) and may be supplemented by FVIII-VWF concentrate. Because most cases of VWD are relatively mild and patients do not suffer from serious spontaneous bleeding, prophylaxis is rarely indicated. Exceptions include patients with type 3 disease plus haemarthroses, severe epistaxis, women with menorrhagia and those with VWD in conjunction with an on-going risk factor for bleeding, such as angiodysplasia.

## Conclusion

Von Willebrand disease (VWD) is the commonest inherited bleeding disorder. However, despite an increasing understanding of the pathophysiology of VWD, the diagnosis of VWD is frequently difficult because of uncertainty regarding the relationship between laboratory assays and function in vivo. The aim of this work is to show that von willebrand disease can be the cause of serious life-threatening hemorrhage and given its prevalence and presenting symptoms, VWD should always be considered in the assessment of children suspected of non-accidental injury, the risk for increased bleeding should be kept in mind when elective and medical procedures are undertaken in this hemostasis disorder.

## Competing interests

The authors declare no competing interest.
